# Four specific biomarkers associated with the progression of glioblastoma multiforme in older adults identified using weighted gene co-expression network analysis

**DOI:** 10.1080/21655979.2021.1975980

**Published:** 2021-09-13

**Authors:** Yushi Yang, Liangzhao Chu, Zhirui Zeng, Shu Xu, Hua Yang, Xuelin Zhang, Jun Jia, Niya Long, Yaxin Hu, Jian Liu

**Affiliations:** a College of Basic Medicine, Guizhou Medical University, Guiyang, Guizhou, China; b Department of Pathology, Guizhou Medical University, Guiyang, Guizhou, China; c Department of Cerebral Surgery, The Affiliated Hospital of Guizhou Medical University, Guiyang, Guizhou, China; d Department of Physical Examination Center, The Affiliated Hospital of Guizhou Medical University, Guiyang, Guizhou, China; e College of Clinical Medicine, Guizhou Medical University, Guiyang, Guizhou, China; f Department of Prenatal Diagnosis, The Affiliated Hospital of Guizhou Medical University, Guiyang, Guizhou, China; g Department of Cerebral Surgery, Guizhou Provincial People’s Hospital, Guiyang, Guizhou, China

**Keywords:** Glioblastoma, elder, biomarkers, WGCNA

## Abstract

Glioblastoma multiforme (GBM) is the most common primary intracranial malignancy in adults. Owing to individual tolerance and tumor heterogeneity, the therapy methods for young adults do not apply to older adults. The present study aimed to identify specific biomarkers for GBM in older adults using weighted gene co-expression network analysis (WGCNA). Gene expression profiles of older adults with GBM were downloaded from The Cancer Genome Atlas (TCGA) and set as a discovery cohort to construct WGCNA. Core genes of clinically significant modules were used to perform functional enrichment, protein-protein interaction, and Pearson correlation analyses. Gene expression profiles of young in TCGA and older GBM patients from our research group were set as verification cohorts for hub gene expression and diagnostic value. Four significant gene modules associated clinically with older adults with GBM were identified, whereas 251 genes were core genes with module membership>0.8 and gene significance>0.2. Ermin (*ERMN*), myelin-associated oligodendrocyte basic protein (*MOBP*), proteolipid protein 1 (*PLP1*), and oligodendrocytic myelin paranodal and inner loop protein (*OPALIN*) genes had significant relationships with the Karnofsky score (KPS) in older GBM patients. *ERMN, MOBP, PLP1*, and *OPALIN* had no relationship with KPS in young GBM patients. These genes were upregulated in GBM tissues from older patients with low but not high KPS and had high diagnostic value. In conclusion, *ERMN, MOBP, PLP1*, and *OPALIN* may serve as specific biomarkers for the progression of GBM in older adults.

## Introduction

Glioblastoma multiforme (GBM) is the most common primary intracranial malignancy in adults, accounting for approximately 70% of all intracranial malignancies [1]. According to various clinical studies, the average overall survival time is less than 15 months. The 2-year survival rate of young GBM patients (age less than 70 years) is 22.2%, whereas it was only 8.3% in older GBM patients (age more than 70 years) [2]. Some studies have shown that the molecular mechanisms involved in the progression of GBM in older adults are inconsistent with those in young adults [3]. Furthermore, because of the poor overall tolerance of the elderly, multiple organ injuries commonly occur during therapy [4]. Therefore, identifying novel biomarkers for GBM in older adults may aid in diagnosis and clinical therapy.

Microarrays and high-throughput sequencing are key technologies for uncovering the landscape of the tumor genome. Bioinformatics analysis of microarray data and high-throughput sequencing data can uncover several novel biomarkers that can aid in clinical diagnosis and therapy [5,6]. In previous studies, numerous valuable mRNAs, miRNAs, and circRNAs, which can contribute to the diagnosis of GBM, have been identified. Zhao *et al*. demonstrated that the ALG13 UDP-N-acetylglucosaminyltransferase subunit gene was dysregulated in GBM, involved in the progression of GBM, and associated with secondary temozolomide-resistance according to microarray data analysis [7]. By analyzing the miRNA microarray, Candido *et al*. showed that miR-29 c was decreased in GBM tissues and could regulate the AKT serine/threonine kinase 3 gene, which is involved in the progression of GBM [8]. Similarly, by analyzing serum circRNA expression profile, Stella *et al*. showed that circ-homeodomain interacting protein kinase 3 had a high diagnostic value for distinguishing GBM tissues from normal tissues [9]. Furthermore, by analyzing gene expression profiles, Zhou *et al*. demonstrated that genes involved in the calcium signaling pathway are associated with the development of GBM [10].

Weighted gene co-expression network analysis (WGCNA) is a valid method for identifying core genes associated with clinical traits and has been widely used to explore biomarkers for cancers in previous studies [11]. In the WGCNA network, genes with high correlations are clustered into a module, and the relationships between gene modules and clinical traits are determined to identify significant modules. The central nodes of the significant modules are regarded as core genes that could play a core role in disease progression. In previous studies, a series of biomarkers were identified via the WGCNA method [12,13]. For example, through WGCNA, Qi Yang *et al*. revealed that six genes (copine 6, hyaluronan and proteoglycan link protein 2, CKLF-like MARVEL transmembrane domain containing 3, N-myc and STAT interactor, capping actin protein gelsolin-like, and proteasome 20S subunit beta 8) were associated with the inflammatory response involved in the progression of GBM [14]. Similarly, Lin *et al*. showed that four genes (transgelin 2, podoplanin, TIMP metallopeptidase inhibitor 1, and epithelial membrane protein 3) are significantly associated with tumor immunology and play key roles in the progression of GBM via WGCNA [15]. However, the latent mechanisms of young and older GBM subtypes are inconsistent; therefore, the feasibility and specificity of the majority of biomarkers identified in previous studies were limited to older GBM patients.

In the present study, we combined WGCNA analysis and related experimental analyses to explore novel biomarkers for GBM in older adults. It was revealed that Ermin (*ERMN*), myelin-associated oligodendrocyte basic protein (*MOBP*), proteolipid protein 1 (*PLP1*), and oligodendrocytic myelin paranodal and inner loop protein (*OPALIN*) were present in the modules associated with Karnofsky performance status (KPS) score and upregulated in the GBM tissues provided by older patients with low KPS scores. This evidence indicates that these four genes could serve as potential biomarkers for GBM in older adults.

## Materials and methods

### Data processing

The gene expression matrix and corresponding clinical characteristics, including survival time, vital status, and KPS score of GBM tissues, were obtained from the Cancer Genome Atlas (TCGA; URL: https://portal.gdc.cancer.gov/) [16]. Patients aged ≥ 70 years were classified as older GBM patients, whereas patients aged <70 years were selected as young GBM patients in the present study. Therefore, the gene expression profiles of 38 GBM tissues provided by older adults were used as the discovery cohort. After annotating probes, removing null probes and low abundance genes, and normalization, 17,851 genes in these 38 tissues were used to construct the WGCNA.

### WGCNA

After checking whether outliers existed via a sample dendrogram, the WGCNA was performed in the R environment (URL: https://www.r-project.org/) [17]. After performing Pearson’s correlation analysis for all gene pairs, a similarity matrix was constructed according to the result. The matrix of similarity was constructed based on appropriate soft power to produce a scale-free co-expression network, which could ensure scale independence ≥ 0.85 and a mean connectivity degree close to 0. The adjacency matrix was then transformed into a topological overlap matrix (TOM). Finally, median linkage hierarchical clustering was analyzed using the TOM-based dissimilarity measure with a minimum size of 50, and the adjacency gene modules with similarity < 0.2 were merged.

### Identification of significant modules and core genes

The relationship between gene modules clustered by WGCNA and the clinical traits of older GBM patients were analyzed. These traits included survival days, vital (dead or alive), and KPS. The thresholds to determine significant module were correlation (R) ≥ 0.3 and P-value < 0.05. In significant modules, gene significance (GS) was determined using associations between the individual genes and the clinical characteristics of interest, along with the module membership (MM), which was determined using the correlation between the module eigengenes and the gene expression profiles. Genes with GS > 0.2 and MM > 0.8 were set as core genes in significant modules for further study.

### Functional enrichment analysis

Functional enrichment analyses of core genes, including Gene Oncology (GO) and Kyoto Encyclopedia of Genes and Genomes (KEGG) analysis, were performed using the online tool Functional Annotation Bioinformatics Microarray Analysis (DAVID; URL: https://david.ncifcrf.gov/) [18]. The terms of enrichment analyses were considered significant at P <0.05.

### Protein-protein interaction (PPI) network

The information on proteins coded by core genes was imported into the STRING database (https://string-db.org/) [19] to obtain their interaction information, and their interaction information was analyzed using Cytoscape software [20]. Nodes in PPI mean proteins and lines indicated the relationship between them. The degree score was an index to reflect the relationship between proteins and was calculated using the Cytohub plug-in (version 1.0; http://github.com/cytoscape/appstore) [21]. Genes with a top 10-degree score were set as hub genes.

### Pearson’s correlation analysis between the hub genes and KPS of GBM patients

The relationship between the hub genes and KPS of GBM patients was further analyzed using Pearson’s correlation analysis in SPSS software (version 20.0). Genes with high correlation (R >0.3 and P <0.05) with KPS of GBM patients were set as real hub genes and used for further analysis.

### Specimen collection

A total of 43 GBM tissues from older patients (years ≥ 70) were obtained from the Pathology Department of the Affiliated Hospital of Guizhou Medical University. Older GBM patients with KPS scores ≥ 60 (n = 28) were classified as the high KPS group, whereas patients with KPS scores < 60 (n = 15) were classified as the low KPS group. None of the patients in the present study underwent chemotherapy or radiation before tissue collection, and all patients provided samples signed written informed consent. The study was approved by the Human Trait Ethics Committee of the Guizhou Medical University.

### Immumohistochemical (IHC) staining

Paraffin-embedded GBM tissues provided by older patients were sectioned at a thickness of 4 μm. All sections were dewaxed and dehydrated using graded concentrations of xylene and alcohol, respectively. After antigen retrieval using citrate buffer (pH 6.0; Zsbio, Beijing, China), the sections were blocked with 5% BSA (Boster, Wuhan, China) and 0.3% H_2_O_2_ to decrease nonspecific binding. The primary antibodies including ERMN (1:200; Cat No. 66,605-1-Ig, Proteintech, Wuhan, China), MOBP (1:100; Cat No.12472-1-AP, Proteintech, Wuhan, China), PLP1 (1:100; Cat No. A20009, Abconal, Wuhan, China) and OPALIN (1:300; Cat. ab121425, Abcam, China), were added to the sections and incubated overnight at 4°C, followed by incubation with anti-mouse (1:2000; cat. no. BM3895) and anti-rabbit (1:2000; cat. no. BM3894) horseradish peroxidase-conjugated goat secondary antibodies (Boster, Wuhan, China) after washing three times with PBS. The sections were then stained with hematoxylin and diaminobenzidine at room temperature. Finally, the images of the sections were obtained using a light orthophoto microscope (magnification ×100). The expression levels of target proteins were evaluated by the sum of the proportion of positively stained cells (0, <1%; 1, 1%–33%; 2, 34%–66%; and 3, 67%–100%) and stain depth (0, no staining; 1, weakly positive; 2, moderately positive; and 3, strongly positive).

### Receiver operating characteristic curve (ROC)

The diagnostic value of real hub genes to distinguish high and low KPS score tissues provided by older adults with GBM was determined using SPSS software via ROC based on the expression score obtained from IHC. Genes with an area under the curve (AUC)>0.7 were considered to possess diagnostic value.

## Results

A total of 23 co-expressed modules were identified in the WGCNA based on data from the TCGA database. Four (cyan, green, lightcyan, and orange) were positively associated with clinical traits, whereas MM was significantly associated with GS. A total of 97, 128, 15, and 11 core genes with MM>0.8 and GS>0.2 were in cyan, green, orange, and lightcyan modules, respectively. These genes were enriched in a series of GO terms and KEGG pathway terms, including vesicle coat and ether lipid metabolism. Among these core genes, *ERMN, MOBP, PLP1*, and *OPALIN* had a high score in the PPI network and were negatively associated with the KPS score in older adults with GBM. Interestingly, *ERMN, MOBP, PLP1*, and *OPALIN* had no relationship with KPS scores in young adults with GBM. Furthermore, we found that *ERMN, MOBP, PLP1*, and *OPALIN* were also highly expressed in GBM tissues obtained from older adults with lower KPS scores compared to those with high KPS scores and had a high diagnostic value in distinguishing the tissues provided from older GBM tissues with high and low KPS scores.

### Constructing the WGCNA

**Figure 1.**
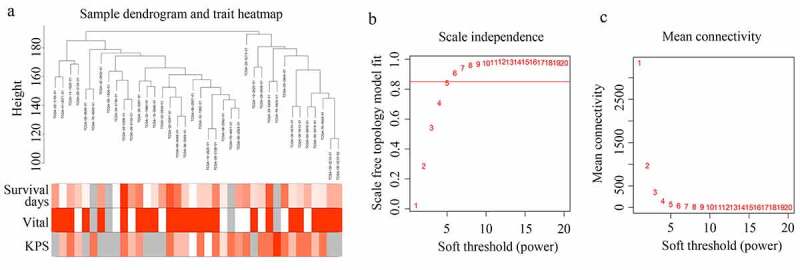
**Structuring WGCNA**. (a) Sample tree clustering and clinical traits (Survival days; vital: white = alive, red = dead, gray = missing value; KPS) heat map of 38 GBM tissues in older adults. (b) Scale independence of various soft-threshold values. (c) Mean connectivity of various soft-threshold values

A sample dendrogram demonstrated that no outliers existed in the GBM tissues provided by older patients in TCGA, and the total gene expression profile of 38 GBM tissues provided by older adults and corresponding traits were used to construct the WGCNA (Figure 1a). As shown in the results (Figure 1b-c), while the soft power β was 6, the scale independence of the topology network reached >0.85, and the mean connectivity was ~ 0. Therefore, the soft power of β = 6 was set as the soft threshold to perform subsequent analyses.

### Significant modules and module core genes were identified in WGCNA

**Figure 2.**
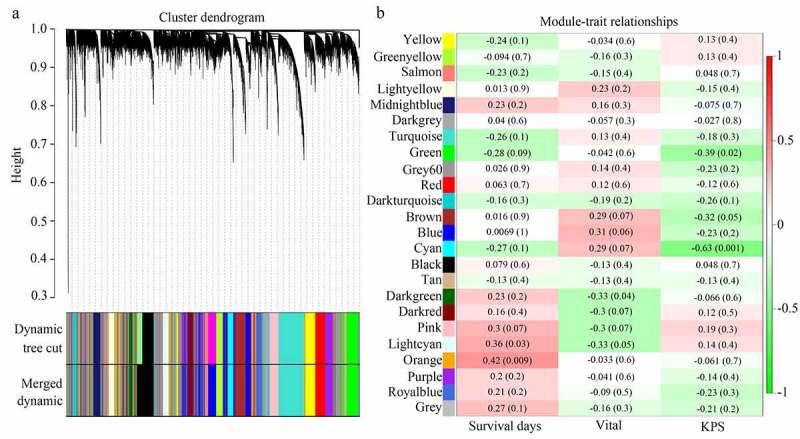
**Module clusters and relationships with clinical traits**. (a) Clustering dendrograms of all genes with dissimilarity based on topological overlap, together with assigned module colors. (b) Identification of significant modules associated with clinical traits (survival days, vital, and KPS). Each cell in the heat map contains the corresponding correlation score and P-value. Red indicates a positive correlation, whereas green indicates a negative correlation

**Figure 3.**
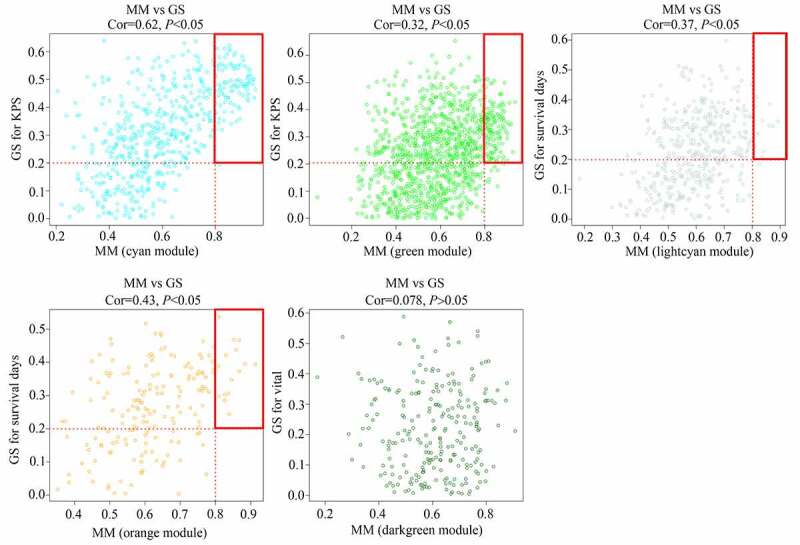
Relationship between gene significance (GS) and module membership (MM) in significant modules

As shown in the results, genes were clustered in 23 co-expressed modules (yellow, greenyellow, salmon, lightyellow, midnightblue, darkgrey, turquoise, green, grey60, red, darkturquoise, brown, blue, cyan, black, tan, darkgreen, darkred, pink, lightcyan, orange, purple, and royalblue), whereas genes that were not co-expressed were all clustered in the gray module (Figure 2a). Among these gene modules, the cyan and green modules were negatively associated with KPS in older adults with GBM (R = −0.63 and R = −0.39, respectively); the orange and lightcyan modules were positively associated with survival days in older adults with GBM (R = 0.42 and R = 0.36, respectively), whereas genes in darkgreen were negatively associated with vital status in older adults with GBM (R = −0.33) (Figure 2b). Then, correlations between GS and MM were calculated for these five modules. We found that the MM of genes in the cyan and green modules were significantly associated with their GS for KPS (cor = 0.62, P <0.05; cor = 0.32, P <0.05). MM of genes in the orange and lightcyan modules were significantly associated with their GS for survival days (cor = 0.43, P <0.05; cor = 0.37, P <0.05). However, the MM of genes in the darkgreen module was not significantly associated with their GS for vital status (cor = 0.078, P >0.05) (Figure 3). Therefore, cyan, green, orange, and lightcyan were set as significant modules were chosen because the thresholds for MM>0.8 and GS>0.2, and there were 97, 128, 15, and 11 in the cyan, green, orange, and lightcyan modules that were selected as core genes. Details of the core genes in each module are shown in Table 1.

**Table 1.** Detail of hub genes in each significant module

Module	Hub gene
Cyan	PPP1R14A DBNDD2 DNAH17 OPALIN HHATL FCHO1 FAM19A4 CLCA4 KIAA1598 ROGDI CAPN3 PLEKHH1 LANCL1 PTGDS S1PR5 LDB3 CD22 TMEM144 CARNS1 TPPP KCNQ1DN LIPE SRCIN1 SPOCK3 SEC14L5 ERMN SLC5A11 MAL SFTPC ADAP1 BOK MBP TMEM63A SLC45A3 CNTN2 AATK ZFP57 CNDP1 NKX6-2 PEX5L CNTNAP4 HAPLN2 CYTH1 LOC283999 KANK4 PLP1 C9orf122 LGI3 TMCC2 GJB1 NKAIN2 QDPR PLCL1 ST18 GPR62 PLCH2 MOBP PCSK6 ENPP2 SEMA4D EDIL3 TMEM151A MAG HCN2 LOC100128675 LOC150622 GJC2 CNP SH3GL3 TMEM125 CDK18 TUBB4 LRP2 SHISA2 LHPP PPP1R16B FA2H MOG C11orf9 C7orf41 KLK6 TMEM88B BCAS1 GAL3ST1 TF RAB40B ZNF536 ZNF488 FAM107B RAB11FIP4 UGT8 CHADL SOX10 GREM1 HOXD1 CLDN11 SLC7A14
Green	DNAH1 HGFAC TRPV1 TFAP2E ATG16L2 JMJD7-PLA2G4B SULT1A3 AGER FAM193B ACCN3 NKTR SCNN1D SGK494 LOC646471 LOC100128288 KAT2A C17orf56 AGAP5 LENG8 DNASE1 C16orf79 PRICKLE4 APBB3 SEC31B KIAA1875 L3MBTL ARID3B SLC26A1 NPIP MYH3 CRIPAK RHOT2 SDHAP3 CEACAM19 STRC NCRNA00176 OSBPL7 NRBP2 HAUS5 PVRIG ATXN7L2 CUL9 TBC1D3B ANKS3 ULK3 FLJ45340 GDPD3 MZF1 CDK5RAP3 TNRC6A KIFC2 SRRM2 PABPC1L PILRB PDXDC2 FAM13AOS TTLL3 ZNF692 TRO LY6G5B LRDD UCKL1AS LOC100132287 CCDC78 CROCCL2 RPL32P3 CRAMP1L YJEFN3 CDK3 NCRNA00107 DND1 ANKRD23 PRDM15 CCDC84 ?|155,060 FBXL6 KLHL17 CSAD WDR90 ?|645,851 LOC100133331 ANKRD36 CCNL2 MSH5 AGAP6 LOC91316 POGZ GSDMB LOC100272228 UCP3 WASH7P DNHD1 PKD1 LOC200030 TTC21A TAF1C C8ORFK29 SPDYE8P CCDC57 GIGYF1 ERAS NCRNA00201 KIAA0895 L UNC5CL ZNF337 NCRNA00105 GNRH1 ASB16 KIAA0907 AFG3L1 ING5 LOC100240726 CDK10 LOC100128842 ANKZF1 LOC349114 HOOK2 STK36 ZNF767 LOC338799 ZNF471 FAM156A LOC150776 AKR7L INE1 FAM71F2 CLK2 TRIM52
Lightcyan	SP3 NEDD1 SAPS3 NUP107 PRPF40A RBM27 NAA15 TMEM194A POLK PRPF4B CDC27
Orange	RC3H2 PAFAH1B2 GTF2A1 SLC30A4 LMBRD2 SBNO1 CCNT1 CRK ETV3 TAOK1 IL6ST NCOA2 LOC284232 MAN1A2 UHMK1

contributing to predicting KPS change.

### Functional enrichment analysis of module core genes

**Figure 4.**
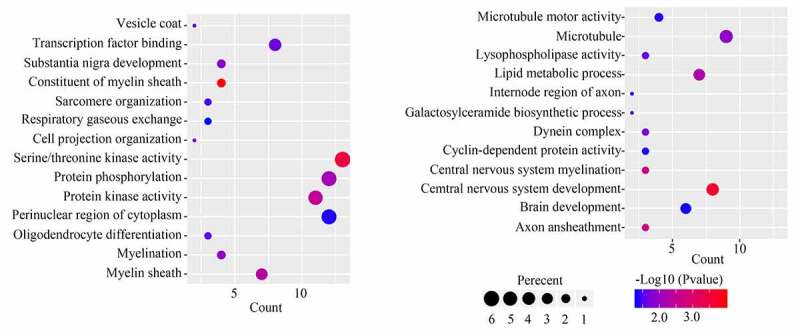
GO analysis for the module core genes

GO analysis for the 251 module core genes showed that these genes were enriched in ‘vesicle coat,’ ‘transcription factor binding,’ ‘substantia nigra development,’ ‘constituent of myelin sheath,’ ‘sarcomere organization,’ ‘respiratory gaseous exchange,’ ‘cell projection organization,’ ‘serine/threonine kinase activity,’ ‘protein phosphorylation,’ ‘protein kinase activity,’ ‘perinuclear region of cytoplasm,’ ‘oligodendrocyte differentiation,’ ‘myelination,’ ‘myelin sheath,’ ‘microtubule motor activity,’ ‘microtubule,’ ‘lysophospholipase activity,’ ‘lipid metabolic process,’ ‘internode region of axon,’ ‘galactosyl ceramide biosynthetic process,’ ‘dynein complex,’ ‘cyclin-dependent protein activity,’ ‘central nervous system myelination,’ ‘central nervous system development,’ ‘brain development’ and ‘axon ensheathment’ (Figure 4). KEGG analysis of the 251 module core genes showed that these genes were enriched in ‘ether lipid metabolism’ and ‘cell adhesion molecules’ pathways.

### PPI network construction

**Figure 5.**
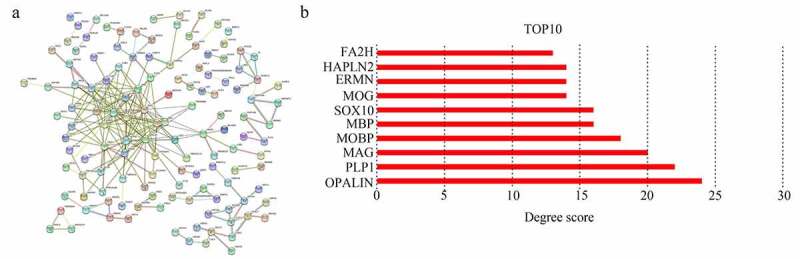
**Selecting hub genes in GBM**. (a) Module core genes used to construct protein-protein interaction network. Nodes indicate genes, lines indicate interactions. (b) Genes with top 10 degree score are shown

A total of 251 module core genes were imported into STRING to construct the PPI network (Figure 5a). The analysis showed that genes [*OPALIN, PLP1*, myelin-associated glycoprotein (*MAG), MOBP*, myelin basic protein (*MBP), SRY-box transcription factor 10* (*SOX10*), myelin oligodendrocyte glycoprotein (*MOG), ERMN*, hyaluronan and proteoglycan link protein 2 (*HAPLN2*), and fatty acid 2-hydroxylase (*FA2H*)] had a top 10 degree score. Therefore, they were identified as hub genes (Figure 5b). Interestingly, these 10 genes were all cyan module genes associated with KPS.

### Pearson correlation analysis for the expression of hub genes and KPS

**Figure 6.**
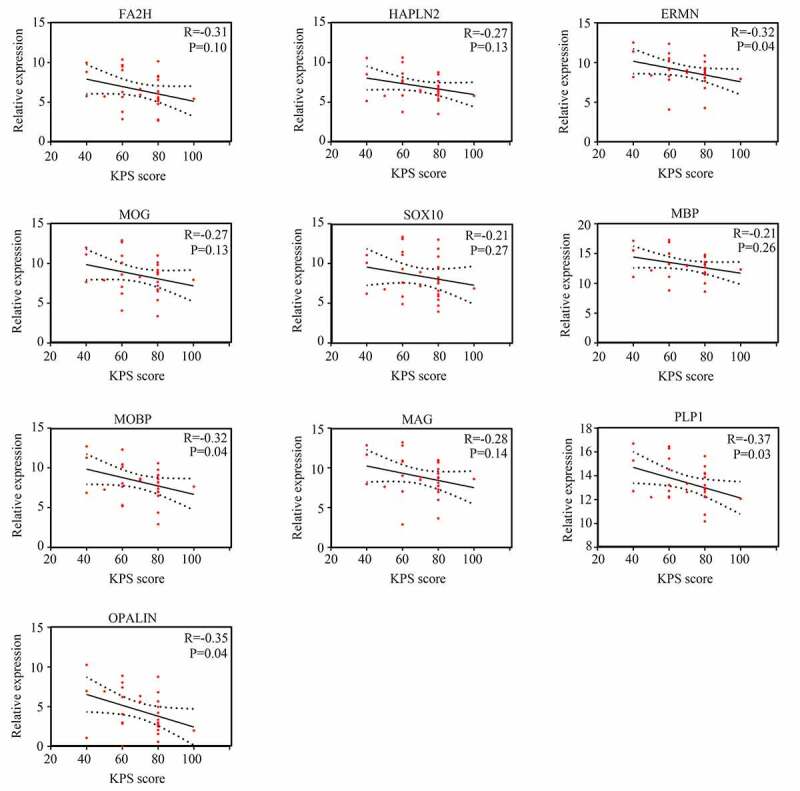
Relationship between the expression of hub genes and KPS score in older patients with GBM

**Figure 7.**
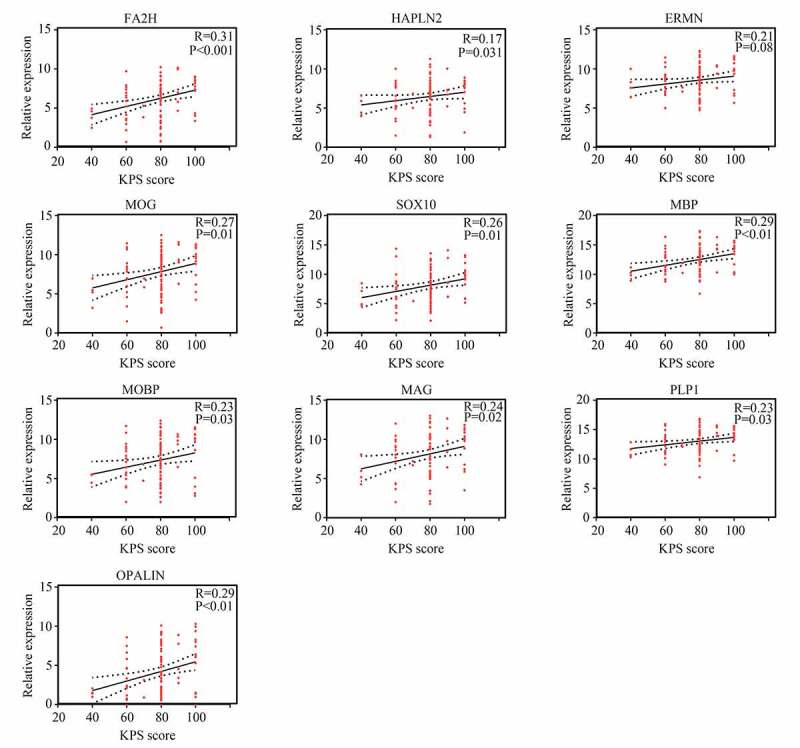
Relationship between the expression of hub genes and KPS score in young patients with GBM

We then used Pearson correlation analysis for the expression of hub genes and KPS in older GBM patients in TCGA. The results showed that expression of *ERMN* (R = −0.32, P <0.05), *MOBP* (R = −0.32, P <0.05), *PLP1* (R = −0.37, P <0.05), and *OPALIN* (R = −0.35, P <0.05) were significantly and negatively associated with KPS in older GBM patients in TCGA, whereas the expression of other hub genes had no significant relationship with KPS in older GBM patients in TCGA (Figure 6). Interestingly, we found that these genes had no significant relationship with KPS in young GBM patients, except for *FA2H* (Figure 7). Therefore, these four genes (*ERMN, MOBP, PLP1*, and *OPALIN*) were set as real hub genes and could have specific functions to predict the KPS in older GBM patients but not in young GBM patients.

***ERMN, MOBP, PLP1*, and *OPALIN* showed high diagnostic value to predict the change of KPS in older GBM patients**

**Figure 8.**
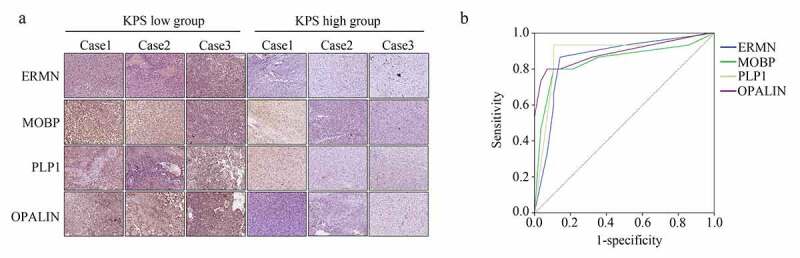
**ERMN, MOBP, PLP1 and OPALIN were highly expressed in the GBM tissues provided by the older patients with lower KPS scores**. (a) IHC stain determined the expression of ERMN, MOBP, PLP1 and OPALIN in GBM tissues provided by the older patients with low and high KPS scores. (b) ROC analysis was performed to determine the diagnostic value of ERMN, MOBP, PLP1 and OPALIN to distinguish the GBM tissues provided by the older patients with low and high KPS scores

We then analyzed the expression of the real hub genes (*ERMN, MOBP, PLP1*, and *OPALIN*) in our verification cohort (43 GBM tissues provided by older patients). IHC results showed that all were upregulated in the GBM tissues provided by the patients with low KPS compared with those provided by the patients with high KPS (Figure 8a). The detailed score of genes in the samples is shown in Table 2. Furthermore, based on the protein level score, we performed ROC analysis, and the results demonstrated that ERMN (AUC = 0.86), MOBP (AUC = 0.846), PLP1 (AUC = 0.88), and OPALIN (AUC = 0.89) all showed high diagnostic value for distinguishing GBM tissues provided from older patients with high and low KPS (Figure 8b).

**Table 2.** Detail IHC score of ERMN, MOBP, PLP1 and OPALIN in GBM tissues provided by the elder patients with low KPS score and high KPS score

Target	Group	IHC score						
		0	1	2	3	4	5	6
ERMN	KPS low	0	1	1	2	2	4	5
	KPS high	0	12	11	2	1	1	1
MOBP	KPS low	0	1	1	1	0	4	8
	KPS high	0	5	14	5	2	1	1
PLP1	KPS low	0	1	0	0	2	4	8
	KPS high	0	11	13	2	0	1	1
OPALIN	KPS low	0	1	1	0	1	3	9
	KPS high	1	17	6	2	1	1	0

## Discussion

Elderly patients with GBM are a unique population. Compared with young patients with GBM, older patients showed a lower overall survival rate and more complications after radiotherapy and chemotherapy [3]. Although various efforts for GBM have been made, there is still no specific treatment method for the elderly population. Therefore, there is an urgent need to uncover the latent mechanism of GBM in older adults and explore novel biomarkers.

In the present study, through the WGCNA in GBM tissues provided from older patients in TCGA, a total of 23 co-expressed modules were clustered. Among them, the cyan and green modules were negatively associated with KPS, whereas the MM of genes in these modules also correlated with GS for KPS; orange and lightcyan modules were positively associated with survival days, and the MM of genes in these two modules also correlated with GS for survival days. A total of 251 core genes were identified in these four modules. Following a series of bioinformatics analyses, including functional enrichment analysis, PPI network construction, and Pearson correlation analysis, four core genes (*ERMN, MOBP, PLP1*, and *OPALIN*) with high degree scores and high correlation with KPS were identified as real hub genes for elderly GBM. Interestingly, these four genes had no significant relationship with KPS in young patients with GBM.

The KPS is a systematic score that evaluates the overall function of a GBM patient [22]. During the progression of GBM, the KPS of patients typically gradually decreases. Furthermore, the treatment options available to patients are increasingly limited [23]. To further analyze the relationship between the four genes (*ERMN, MOBP, PLP1*, and *OPALIN*) and KPS in older adults with GBM, 28 GBM tissues were provided by older adults with GBM with KPS ≥ 60 and 15 GBM tissues provided by older adults with GBM with KPS < 60 were set as the verification cohort. Our results indicated that all four genes were upregulated in the tissues provided by low KPS group patients and had high diagnostic value to distinguish the GBM tissues provided from older patients with high and low KPS. This is the first evidence that ERMN, MOBP, PLP1, and OPALIN may be novel biomarkers for GBM in older adults, which may also have the potential to predict changes in KPS.

The *ERMN*-encoded protein is a cytoplasmic protein located in the outer tongue of the myelin sheath and the paranodal loops of oligodendrocytes. It plays a key role in the formation of myelin sheaths [24]. ERMN expression is dysregulated in a series of nervous system diseases, including multiple sclerosis [25] and neurodegenerative disorders [26]. Similarly, a previous study demonstrated that *ERMN* was highly expressed in the microenvironment of prostate adenocarcinoma and had the potential to regulate the tumor immune response [27]. This evidence suggested that *ERMN* affected the KPS score in elder patients with GBM via regulating tumor immune response. MOBP is also a component of the myelin sheath, which can stabilize the myelin sheath by binding the negatively charged acidic phospholipids of the cytoplasmic membrane [28]. High MOBP expression is significantly associated with the occurrence of encephalomyelitis [29]. Interestingly, MOBP was upregulated in male smokers with lung cancer [30]. However, the molecular mechanism of MOBP in cancers, including GBM, remains unknown. *PLP1* encodes a protein with 276 amino acids, which is the most abundant myelin protein in the brain and can regulate myelin lamellar spacing/compaction and maintain axonal integrity via oligodendrocyte-axonal interactions [31]. Previous studies revealed that overexpression of PLP1 enhanced the accumulation of lipids in the myelin sheath, thereby promoting the progression of diseases in the central nervous system, such as Pelizaeus-Merzbacher disease [32,33]. Furthermore, PLP1 has the potential to inhibit the endoplasmic reticulum [34]. Given the evidence that the endoplasmic reticulum is a key process that can induce cell apoptosis in GBM [35], we consider that PLP1 is involved in the progression of elderly patients with GBM by inhibiting the processes of the endoplasmic reticulum. *OPALIN-*encoded protein is a transmembrane sialoglycoprotein located in the myelin paranodal loop membrane [36]. *OPALIN* induces oligodendrocyte differentiation [37]. *OPALIN* decreased the progression of hereditary spastic paraplegia [38]. *OPALIN* is also involved in cerebral neuroprotection during doxorubicin chemotherapy by decreasing the rate of drugs crossing the blood-brain barrier [39]. We speculated that OPALIN might be involved in the process of patients with GBM via these effects. However, until now, there has been no evidence revealing the role of OPALIN in GBM. Furthermore, although we showed that *ERMN, MOBP, PLP1*, and *OPALIN* might be novel biomarkers for GBM in older adults via the WGCNA and relative experiments, their molecular mechanisms in GBM in older adults should be determined with additional experiments.

## Conclusions

In summary, through WGCNA and relative experiments, we showed that *ERMN, MOBP, PLP1*, and *OPALIN* were core genes in the modules significantly associated with KPS in elderly GBM patients. High expression of *ERMN, MOBP, PLP1*, and *OPALIN* was associated with low KPS in elderly patients with GBM. However, *ERMN, MOBP, PLP1*, and *OPALIN* had no significant relationship with KPS in young patients with GBM. They were all upregulated in the tissues provided by the low KPS group of older GBM patients compared with that provided by the high KPS group and had high diagnostic value to distinguish the GBM tissues provided from older patients with high and low KPS. *ERMN, MOBP, PLP1*, and *OPALIN* may be novel and specific biomarkers for GBM in older adults,

## Data Availability

The data profile of GBM in older adults and the corresponding clinical traits were downloaded from the TCGA database (https://portal.gdc.cancer.gov/). For the verification cohort, the IHC staining results for the 43 GBM tissues provided from older patients can be obtained from the corresponding author upon request.

## Research highlights

- Twenty-three co-expressed modules were identified in GBM tissues from the elderly
- Four gene modules were positively associated with clinical traits
- *ERMN, MOBP, PLP1*, and *OPALIN* were associated with KPS scores in older GBM patients
- *ERMN, MOBP, PLP1*, and *OPALIN* were not related to KPS scores in young GBM patients
- *ERMN, MOBP, PLP1*, and *OPALIN* related to elderly patients and had diagnostic value
